# First report on the phylogenetic relationship, genetic variation of *Echinococcus shiquicus* isolates in Tibet Autonomous Region, China

**DOI:** 10.1186/s13071-020-04456-w

**Published:** 2020-11-23

**Authors:** Guo-Qiang Zhu, Hong-Bin Yan, Li Li, John Asekhaen Ohiolei, Yan-Tao Wu, Wen-Hui Li, Nian-Zhang Zhang, Bao-Quan Fu, Wan-Zhong Jia

**Affiliations:** 1 State Key Laboratory of Veterinary Etiological Biology/National Professional Laboratory for Animal Echinococcosis/Key Laboratory of Veterinary Parasitology of Gansu Province/Key Laboratory of Zoonoses of Agriculture Ministry, Lanzhou Veterinary Research Institute (CAAS), Lanzhou, 730046 Gansu People’s Republic of China; 2 Jiangsu Co-innovation Center for Prevention and Control of Important Animal Infectious Disease, Yangzhou, 225009 Jiangsu People’s Republic of China

**Keywords:** *Echinococcus shiquicus*, Epidemiology, Genetic diversity, Tibet region

## Abstract

**Background:**

Cystic or alveolar echinococcosis caused by the larval stages of *Echinococcus* spp. is a very severe zoonotic helminth infection. *Echinococcus shiquicus* is a newly discovered species that has only been reported in the Qinghai and Sichuan provinces of the Qinghai-Tibet plateau, China where, to date, it has only been confirmed in Tibetan foxes and wild small mammal populations of the Tibetan plateau. Information on its genetic and evolutionary diversity is scanty. The aim of this study was to investigate the prevalence of *E. shiquicus* in plateau pikas (*Ochotona curzoniae*), a known intermediate host, and to determine the genetic variation and phylogenetic relationship of the *E. shiquicus* population in the Tibet region of China based on mitochondrial DNA.

**Methods:**

*Echinococcus shiquicus* samples were collected from Damxung and Nyêmo counties (located in Tibet Autonomous Region, China). The mitochondrial *cox*1 and *nad*1 gene sequences were analyzed, and the genetic diversity and epidemiology of *E. shiquicus* in the region were discussed based on the results.

**Results:**

The prevalence of *E. shiquicus* in pikas in Damxung and Nyêmo counties was 3.95% (6/152) and 6.98% (9/129), respectively. In combination with previous public sequence data, the haplotype analysis revealed 12 haplotypes (H) characterized by two distinct clusters (I and II), and a sequence distance of 99.1–99.9% from the reference haplotype (H1). The diversity and neutrality indices for the entire *E. shiquicus* populations were: haplotype diversity (Hd) ± standard deviation (SD) 0.862 ± 0.035; nucleotide diversity (Hd ± SD) 0.0056 ± 0.0003; Tajima's* D* 0.876 (*P* > 0.05); and Fu’s* F* 6.000 (*P* > 0.05).

**Conclusions:**

This was the first analysis of the newly discovered *E. shiquicus* in plateau pikas in the Tibet Autonomous Region of China. The neutrality indices suggest a deficiency of alleles, indicative of a recent population bottleneck.

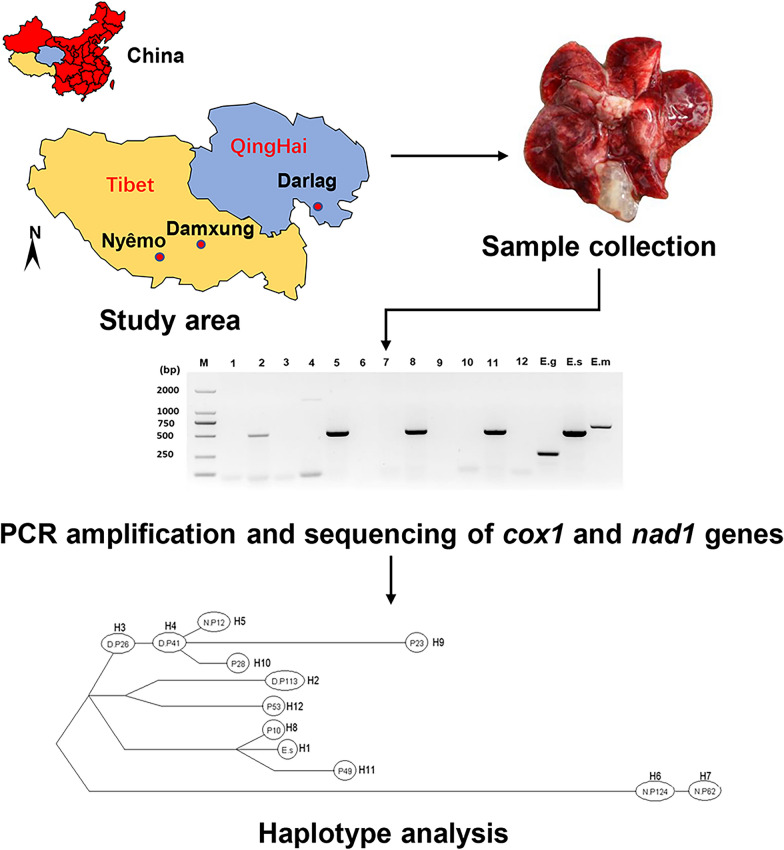

## Background

Echinococcosis or hydatidosis is a serious cosmopolitan parasitic zoonosis [1–3]. *Echinococcus shiquicus*, a newly described species of the genus *Echinococcus*, is currently believed to be limited and endemic to the Qinghai-Tibet plateau region of China [4–6] where the Tibetan fox (*Vulpes ferrilata*) and plateau pika (*Ochotona curzoniae*) commonly serve as the definitive and intermediate hosts, respectively. *E. shiquicus* was also recently identified in some rodent species, such as voles, in Sichuan province, which further suggests other small mammal species could serve as potential intermediate hosts to this *Echinococcus* species [7]. Also, following the detection of *E. shiquicus* DNA molecules in dog faeces in Sichuan province [7], there are concerns that dogs could have the potential to serve as a definitive host for this helminth. Taking into account the close proximity of dogs to humans and the limited knowledge currently available the zoonotic potential of *E. shiquicus*, this helminth represents a significant public health concern. However, further study is needed to determine whether this concern is valid.

Since the discovery of this species, several questions have been raised: Is the infection caused by this species zoonotic? What are its epidemiology and geographical distribution? What is the overall difference between this species and other species of *Echinococcus*? What is its possible economic impact? What is its degree of endemicity? Why does it appear to be geographically restricted to the Qinghai-Tibet Plateau? [5, 8]. The answers to these questions will rely on the accumulation of data over time across the Qinghai-Tibet Plateau.

Tibetan pastoral communities remain the most *Echinococcus* multi-species endemic regions in China [9]. A major challenge in the past to the study of *Echinococcus* species was the common misidentification of *E. shiquicus* as *E. multilocularis* due to a similar transmission pattern and host range; detailed knowledge of its morphological structure eventually resolved this problem [10]. However, there remains a dearth of information on the epidemiological status of *E. shiquicus*, even though the problem of misidentification has been resolved with the development and implementation of specific and reliable methods for differentiating species of *Echinococcus* [11, 12]. Also, the rapid development in sequencing technology, specific genetic markers, especially mitochondrial (mt) DNAs and the development of useful tools/software for resolving the evolutionary and phylogenetic relationships between and within species [13–16] will further benefit our understanding of the phylogenetic relationship existing among *E. shiquicus*.

Here, we report for the first time *E. shiquicus* in plateau pikas (*Ochotona curzoniae*) in the Tibet Autonomous Region (Nyêmo and Damxung counties), China. We have estimated its genetic variation and evolutionary relationship using concatenated *cox*1*–nad*1 nucleotide sequence (2505 bp).

## Methods

### Collection of samples

**Fig. 1:**
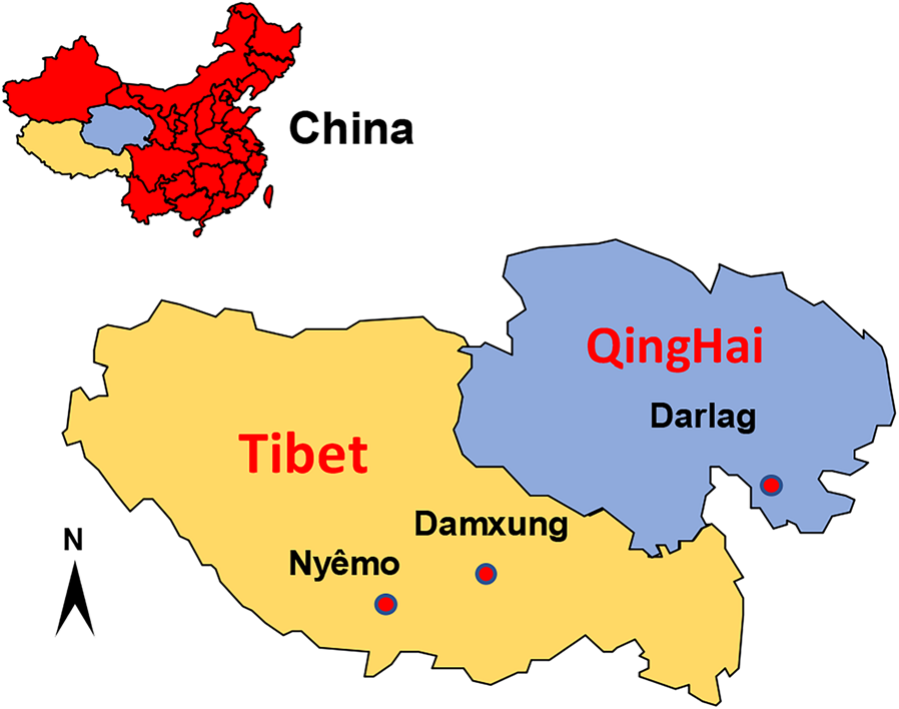
Map showing Damxung and Nyêmo counties in the Tibet Autonomous Region (yellow) and Darlag County in Qinghai (blue).

A total of 281 plateau pikas were trapped in Damxung county (*n* = 152) (91° 1ʹ 42ʺ E, 30° 29ʹ 3ʺ N; altitude elevation 4360 m a.s.l.) and Nyêmo county (*n* = 129) (90° 17ʹ 2ʺ E, 29° 37ʹ 54ʺ N; altitude elevation 4640 m a.s.l.) in China (Fig. 1) using break-back traps set at the entrance of their dens. All internal organs, including the heart, liver, spleen, lung and kidney, were examined by necropsy for cysts. All cystic-like samples (see Table 2 for cyst location) were examined morphologically on the spot before transportation to the State Key Laboratory at Lanzhou Veterinary Research Institute, CAAS, for molecular identification and further analysis. All samples were stored in liquid nitrogen until DNA extraction.

### DNA extraction and identification of *Echinococcus* species

Before DNA extraction, samples were removed from liquid nitrogen and placed in an icebox (containing ice) to thaw. A sample of tissues (about 10 mg) of each cyst was cut off and placed in a 1.5-ml micro-centrifuge tube following which it was ground manually with a pestle for 2 min on ice before being digested with proteinase K. Genomic DNA was extracted using the DNeasy Blood and Tissue Kit according to the manufacturer's instructions (Qiagen, Hilden, Germany). The DNA concentration was measured on a microplate reader (Infinite® 200 PRO NanoQuant; Tecan Group Ltd., Männedorf, Switzerland) and then stored at − 20 °C until use.

### Amplification and sequencing of *Echinococcus shiquicus cox*1 and *nad*1 genes

A multiplex PCR assay previously described [12] was initially used to confirm the species (*E. granulosus s.s.*, *E. multilocularis* and *E. shiquicus*) responsible for the infection. Afterward, the complete *nad*1 and *cox*1 genes of *E. shiquicus* from all positive samples were further amplified using specific primers (Table 1) that were designed based on the complete mtDNA sequence of *E. shiquicus* (GenBank accession no. AB208064 or NC_009460). PCR amplification was carried out in a 50-μl reaction volume (25 μl Premix *Taq*™ [Takara Bio, Kusatsu, Japan], 1 μl forward and reverse primers [1 μM of each primer] and 1 μl of extracted DNA [100 μg/ml]). The PCR reaction was performed under the following conditions: initial denaturation at 95 °C for 3 min; 35 cycles of denaturation at 95 °C for 30 s, annealing at 55 °C for 30 s, and extension at 72 °C, for 90 s; followed by a final extension at 72 °C for 10 min. Gel electrophoresis was used to detect PCR products in a 1.5% (w/v) agarose gel stained with GelRed® and visualized under UV light (Molecular Imager® ChemiDoc™ XRS+ with Image Lab™ software; Bio-Rad, Hercules, CA, USA). PCR products were then sequenced (TSINGKE Biological Technology Company, Xi’an, China) using the same primer sets.

**Table 1:** Forward and reverse PCR primers used for complete analysis of *Echinococcus shiquicus cox*1 and *nad*1 genes based on complete mitochondrial DNA sequence of *E. shiquicus* (NC_009460 or AB208064)

Primer name	Primer sequences 5ʹ–3ʹ	Size (bp)	Position in the mtDNA^a^
E.s-*cox*1-F	AGTTACTGCTAATAATTTTGTGTCAT	1837	9222
E.s-*cox*1-R	ATGATGTAAAAGGCAAATAAACC		10,829
E.s-*nad*1-F	TAATGTTGATTATAGAAAATTTTCGTTTTACACGC	1286	7520
E.s-*nad*1-R	CACAATTTATTATATCAAAGTAACCTGC		8416

*E.sE. shiquicus*, *F* forward primer,* mt* mitochondrial, *R* reverse primer

^a^Location of the primers

### Phylogenetic relationship and genetic variation analyses

The EditSeq software module of DNAstar software (DNASTAR, Madison, WI, USA) was used to manually edit, align and find open reading frame (ORF) sequences of the complete *nad*1 and *cox*1 genes. Based on the concatenated *cox*1–*nad*1 genes, the phylogenetic tree was generated using the software tool Mrbayes v3.1.2 with settings run for 2,500,000 generations, ensuring that the average standard deviation of split frequency was < 0.01 (0.009577) [17]. The concatenated sequence of the *E. multilocularis cox*1 and *nad*1 genes (GenBank accession no. AB385610 and AB668376, respectively) was considered as an outgroup [6, 10, 18]. In addition, the *cox*1-*nad*1 sequence of *E. shiquicus* (AB208064 or NC_009460) was chosen as a reference sequence while sequences from Fan et al. [18] were included in our analysis to provide better insight into the genetic diversity of *E. shiquicus* in the plateau region as this study is the sole study to date to have investigated the genetic diversity of *E. shiquicus* using longer mitochondrial DNA sequences (2505 bp). The genetic diversities and distances of the concatenated sequences of *E. shiquicus* were inferred by the haplotype network of TCS 1.21 and MegAlign software [19, 20], respectively, while the number of haplotypes, population diversity and neutrality indices were calculated using DnaSP 5.10 software [21].

## Results

### *Echinococcus shiquicus* prevalence and identification

**Fig. 2:**
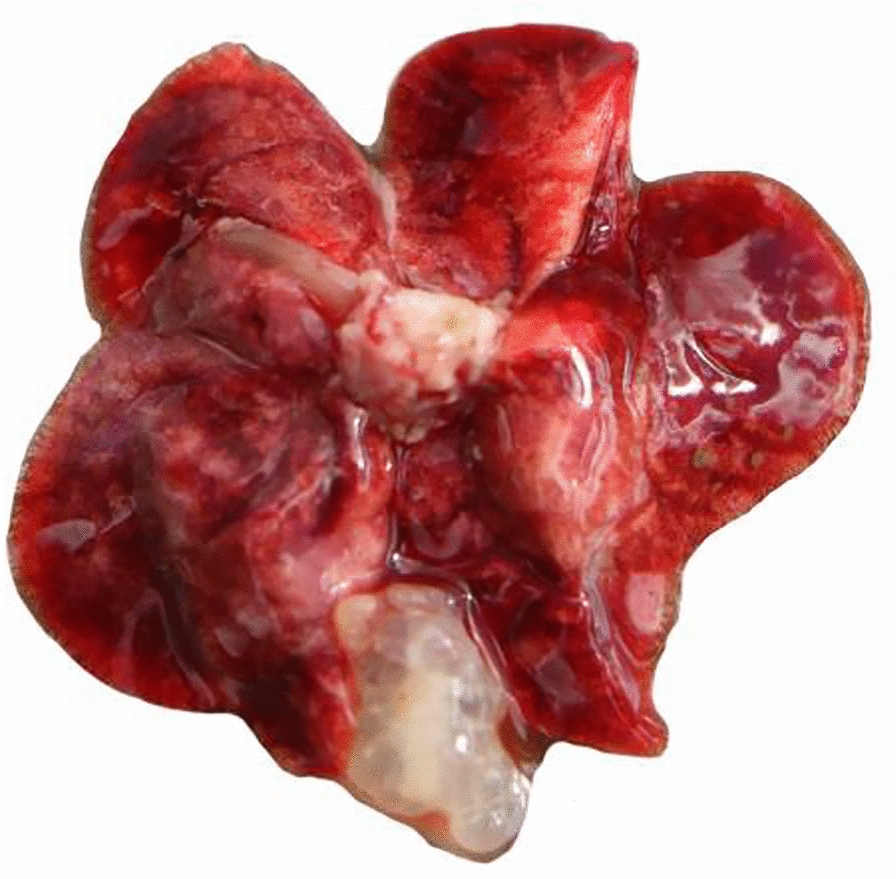
An *Echinococcus shiquicus* isolate from the lung of plateau pikes (*Ochotona curzoniae*), in the Tibet Autonomous Region, China

The prevalence of *E. shiquicus* in plateau pikes in the present study areas, Damxung and Nyêmo counties, was 3.95% (6/152) and 6.98% (9/129), respectively. The multiplex assay revealed that all positive amplified samples were of *E. shiquicus* expected size (471 bp); no *E. multilocularis*- and *E. granulosus*-specific bands were produced or observed. These results confirm that infection was caused by *E. shiquicus* only (Additional file 1: Fig. S1). Most isolates were collected from the lungs (Fig. 2), with a few collected from the spleen, kidney and other tissues (Table 2).

**Table 2:** Characteristics of *E. shiquicus* isolates

Sample number	Sample ID	Infected organ or tissue	Location of collection	Haplotype	Sequence distance from H1 (%)	References
1	ref. isolate	Lung	Sichuan	H1	–	Yuan et al. [9]
2	D.P113	Lung	Damxung	H2	99.6	This study
3	D.P149	Lung	Damxung	H2	99.6	This study
4	D.P151	Chest cavity	Damxung	H2	99.6	This study
5	D.P2	Lung	Damxung	H2	99.6	This study
6	D.P26	Abdomen	Damxung	H3	99.8	This study
7	D.P41	Chest	Damxung	H4	99.7	This study
8	N.P12	Lung	Nyêmo	H5	99.7	This study
9	N.P124	Lung	Nyêmo	H6	99.1	This study
10	N.P15	Lung	Nyêmo	H4	99.7	This study
11	N.P43	Lung	Nyêmo	H2	99.6	This study
12	N.P62	Lung	Nyêmo	H7	99.1	This study
13	N.P64	Lung	Nyêmo	H6	99.1	This study
14	N.P72	Lung	Nyêmo	H6	99.1	This study
15	N.P83	Lung	Nyêmo	H6	99.1	This study
16	N.P89	Lung	Nyêmo	H6	99.1	This study
17	P10	Lung	Darlag	H8	99.9	Cai et al. [18]
18	P23	Lung	Darlag	H9	99.5	Cai et al. [18]
19	P27	Lung	Darlag	H6	99.1	Cai et al. [18]
20	P28	Lung	Darlag	H10	99.6	Cai et al. [18]
21	P29	Lung	Darlag	H8	99.9	Cai et al. [18]
22	P31	Lung	Darlag	H9	99.5	Cai et al. [18]
23	P32	Lung	Darlag	H6	99.1	Cai et al. [18]
24	P34	Lung	Darlag	H9	99.5	Cai et al. [18]
25	P45	kidney	Darlag	H6	99.1	Cai et al. [18]
26	P49	Lung	Darlag	H11	99.8	Cai et al. [18]
27	P50	Lung	Darlag	H9	99.5	Cai et al. [18]
28	P52L	Lung	Darlag	H6	99.1	Cai et al. [18]
29	P52S	Spleen	Darlag	H6	99.1	Cai et al. [18]
30	P53	Lung	Darlag	H12	99.6	Cai et al. [18]
31	P54	Lung	Darlag	H12	99.6	Cai et al. [18]
32	P55	Lung	Darlag	H9	99.5	Cai et al. [18]
33	P6	Lung	Darlag	H9	99.5	Cai et al. [18]
34	P7	Lung	Darlag	H9	99.5	Cai et al. [18]
35	P70	Lung	Darlag	H8	99.9	Cai et al. [18]
36	P77	Lung	Darlag	H11	99.8	Cai et al. [18]

To investigate the genetic diversity, we analysed a total of 35 *E. shiquicus* nucleotide sequences, including sequences representing the 15 isolates from the current study [Damxung (6) and Nyêmo (9) counties of the Tibet Autonomous region] and 20 complete *cox*1-*nad*1 sequences from our previous investigation in Darlag county, Qinghai province [6].

### Haplotypes and phylogenetic tree

**Fig. 3:**
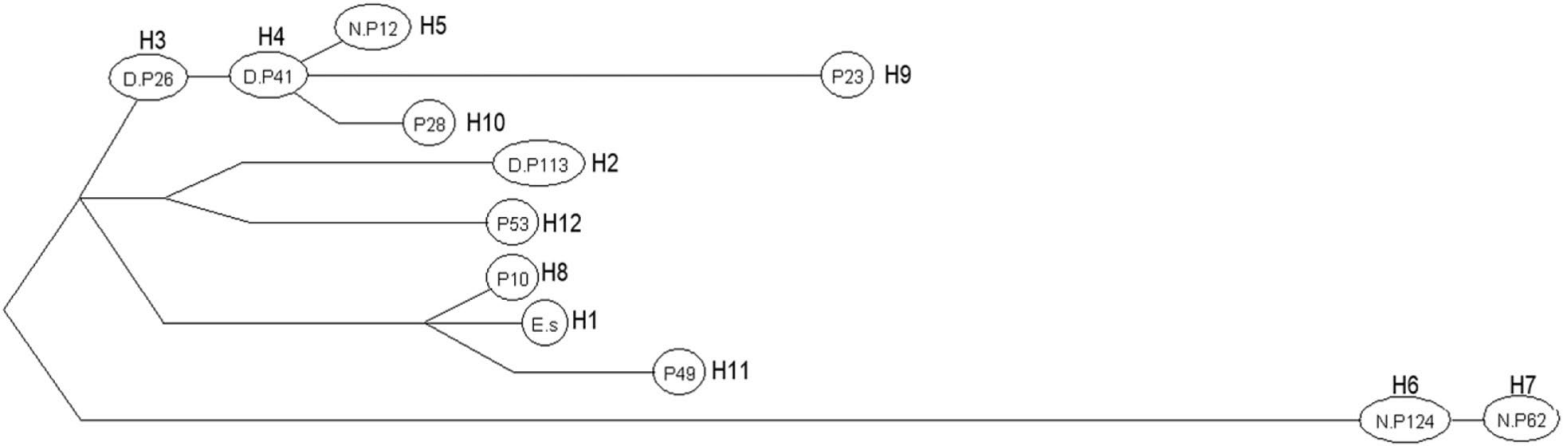
The haplotype network and distribution of the different haplotypes. Letters in circles refer to the sample ID (see Table 2).* H* Haplotype

**Fig. 4:**
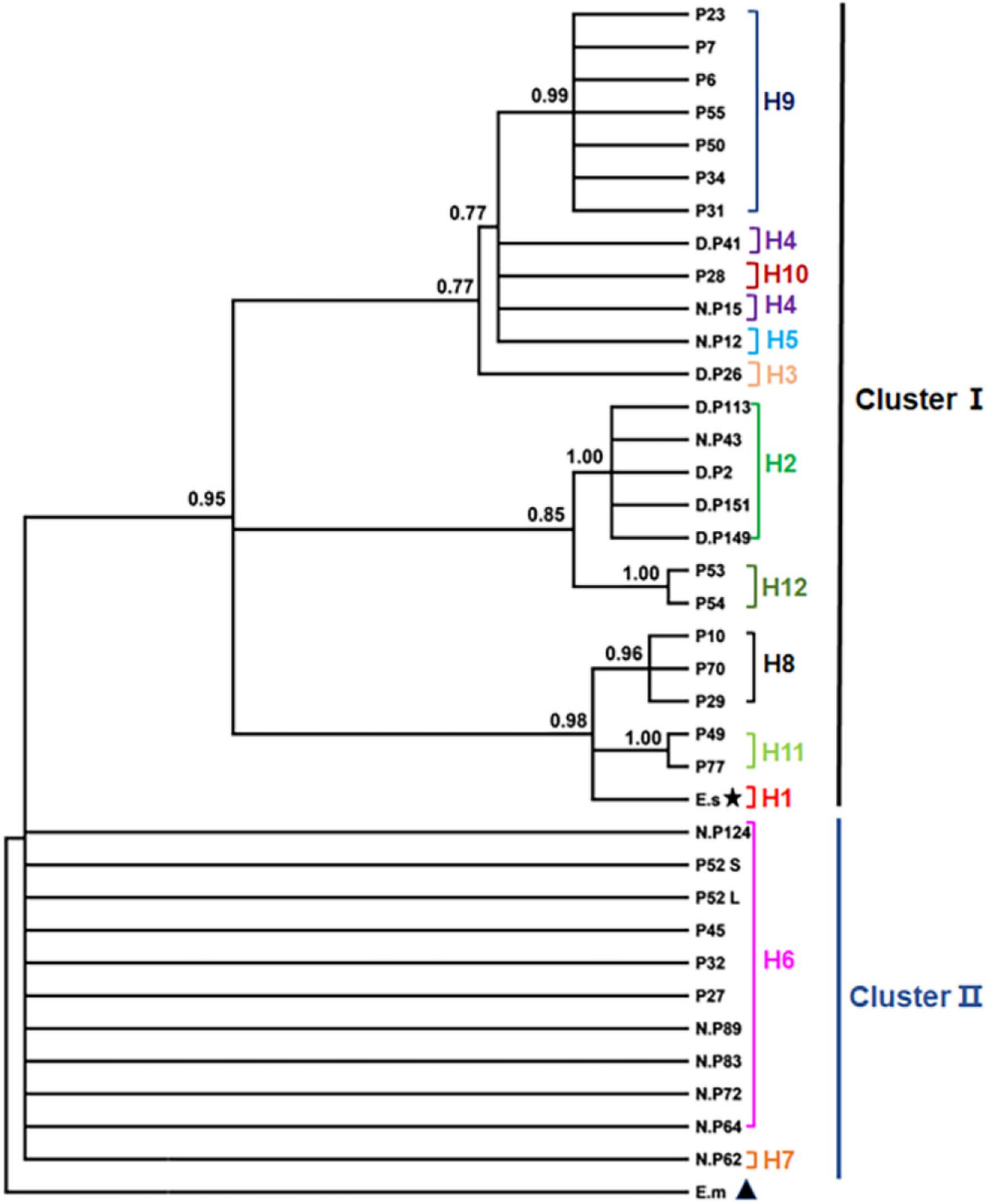
Phylogenetic inference and cluster distribution of *E. shiquicus* isolates based on the concatenated *cox*1–*nad*1 genes.* E.m* *E. multilocularis*, *E.s E. shiquicus*; see Table 2 for other sample ID. (★ = *E*. *shiquicus* reference sequence, ▲ = *E*. *multilocularis* reference sequence)

The entire sequences of *cox*1 and *nad*1 were amplified and sequenced using *E. shiquicus*-specific primers (Table 1) with a PCR product size of 1837 and 1286 bp, respectively. Using the concatenated ORF sequence of the *cox*1 and *nad*1 genes (2505 bp), the *E. shiquicus* isolates formed 11 different haplotypes (H2–H12) (Table 2) in addition to the *E. shiquicus* reference sequence (H1) (GenBank accession no. AB208064 or NC_009460). Of these 11 haplotypes, haplotypes H2–H7 represent haplotypes from this study while haplotypes H6 and H8–H12 represent haplotypes from samples collected in Darlag County. The relationships between these haplotypes and their genetic diversities are shown in Table 2 and Fig. 3, respectively. When compared with the reference sequence (H1), we found H6 to be the farthest genetically distant haplotype from H1 (99.1%) and H8 the closest (99.9%) (Table 2). The Bayesian phylogenetic tree produced two distinct clusters (I and II) of the *E. shiquicus* population (Fig. 4), with a 0.6% difference in the average genetic distance between cluster I (99.7%) and II (99.1%).

### Diversity and neutrality indices

Diversity and neutrality indices for *E. shiquicus* isolates in each location (Darlag, Nyêmo and Damxung) were calculated using the concatenated *cox*1-*nad*1 sequences (Table 3). The total number of mutations was 47, and the overall value of haplotype diversity and nucleotide diversity were 0.862 ± 0.035 and 0.0056 ± 0.0003, respectively (Table 3). Haplotype and nucleotide diversities of isolates from Darlag were higher than those from Nyêmo and Damxung. Neutrality indices calculated by Tajima's* D* (0.876; *P* > 0.05) and Fu’s* F* (6.000; *P* > 0.05) were positive and non-significant, suggesting allele deficiency and a recent population bottleneck event of the *E. shiquicus* population in the Tibetan region.

**Table 3:** Diversity and neutrality indices for *E. shiquicus* populations in the Tibet Autonomous region

Location	Diversity					Neutrality^a^	
	Number of isolates	Total number of mutations	Number of haplotypes	Hd ± SD	Nd ± SD	Tajima's* D* (*P* value)	Fu’s* F* (*P *value)
Damxung	6	7	3	0.600 ± 0.215	0.0014 ± 0.0005	0.888 (0.814)	2.161 (0.882)
Nyêmo	9	27	5	0.722 ± 0.159	0.0045 ± 0.0012	0.703 (0.787)	3.424 (0.932)
Darlag	20	41	6	0.811 ± 0.055	0.0057 ± 0.0006	0.894 (0.850)	8.814 (0.998)
Overall	35	47	11	0.862 ± 0.035	0.0056 ± 0.0003	0.876 (0.867)	6.000 (0.965)

*Hd* Haplotype diversity, *Nd* nucleotide diversity, *SD* standard deviation

^a^Neutrality indices calculated by Tajima's* D* and Fu’s* F* were positive and non-significant (*P* > 0.05)

## Discussion

The geographical distribution, epidemiology and zoonotic potential of *E. shiquicus* remains poorly understood [5]. Although initially reported to be limited geographically to the Qinghai-Tibet Plateau, its presence has since been gradually found in other areas of the plateau [6], thereby increasing the need to intensify efforts to learn more about this species, especially its prevalence, genetic population structure, and host range, in order to understand its transmission pattern and ultimately achieve control of its spread. The use of improved specific detection and discrimination assays [10, 11] in the differential diagnosis of *Echinococcus* infection in the Qinghai-Tibet plateau cannot be overstated in terms of providing a near-accurate picture of the prevalence of the species in the region. For example, the challenge posed by misidentification was recently discussed by Boufana et al. who investigated the possible presence of *E. shiquicus* in hosts other than Tibetan foxes and pikas and found for the first time since the discovery of this helminth that dogs could potentially serve as a definitive host for this species [7]. These authors also observed a co-infection with *E. shiquicus* in a previously identified *E. granulosus* sample and identified cross-reaction of primers as a major reason for missing the *E. shiquicus* in the first instance [7].

The results of this study showed a prevalence of *E. shiquicus* infection in pikas in Damxung and Nyêmo counties of 3.95 and 6.98%, respectively. With the question of the zoonotic potential of *E. shiquicus* still unanswered [5, 22], the prevalence of *E. shiquicus* infection observed in the study area and the prevalence of 23.75–37.5% previously reported in Darlag and Shiqu counties of Qinghai and Sichuan provinces, respectively [2, 6, 23], raises concern regarding the significance of *E. shiquicus* infection for both humans and livestock in the region.

Earlier studies have raised the controversial point of whether or not *E. shiquicus* has evolved without bottleneck effect [13, 24]. However, the neutrality test in the current study suggests a deficiency of alleles which otherwise indicates that the Tibetan *E. shiquicus* population could have possibly experienced a bottleneck effect in the course of evolution.

Among the 11 haplotypes identified in sequences reported previously from Qinghai province [6], H2 and H4 were found in Tibet counties and H6 was common to counties in Qinghai and Tibet; however, no haplotype was found to be common to all the three counties. Our results are in agreement with previous observations demonstrating the absence of a common haplotype across different locations in the plateau [13, 24].

In this study, we report the genetic diversity and the phylogenetic status of *E. shiquicus* population from Tibet in comparison to previous isolates from Qinghai province. Our results demonstrate allele deficiency among the *E. shiquicus* population indicating a population bottleneck event in the Tibet Autonomous Region. However, we suggest that future studies on population genetics consider using a larger panel of isolates and examining other genetic markers in order to further elucidate the evolutionary status of *E. shiquicus* in the Qinghai-Tibet Plateau.

## Conclusions

This study demonstrates the existence of *E. shiquicus* in plateau pikas in the Tibet Autonomous Region of China, and the neutrality indices suggest a deficiency of alleles as expected from a recent population bottleneck.

## Supplementary information

**Additional file 1: Fig. S1.** Multiplex PCR results image of *Echinococcus shiquicus* samples based on three specific primers of *E. multilocularis*, *E. shiquicus* and *E. granulosus.*

## Data Availability

All data supporting the conclusions are included in the article. Representative nucleotide sequences of *cox*1 and *nad*1 genes from the present study are available in the GenBank database under the accession numbers (MW072804-MW072815).
