# Supplementary material for: First report on the phylogenetic relationship, genetic variation of Echinococcus shiquicus isolates in Tibet Autonomous Region, China
Source: Parasit Vectors. 2020 Nov 23;13:590. doi: 10.1186/s13071-020-04456-w (PMC7686673; doi:10.1186/s13071-020-04456-w)
Supplement: Supplementary file 1 — Additional file 1: Fig. S1. Multiplex PCR results image of Echinococcus shiquicus samples based on three specific primers of E. multilocularis, E. shiquicus and E. granulosus. [file 13071_2020_4456_MOESM1_ESM.docx]

Additional file

**Fig. S1** The PCR results of partial *Echinococcus shiquicus* samples by three specific primers of *E. multilocularis*, *E. shiquicus* and *E. granulosus*

*
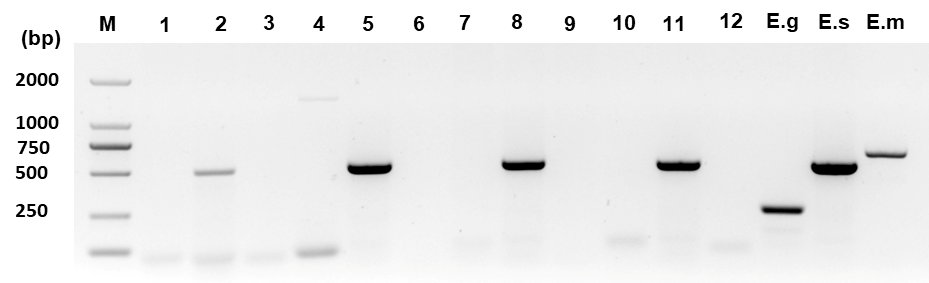
*

M: DL2000 DNA Marker; E.g, E.s and E.m are positive control of *E. granulosus*, *E. shiquicus* and *E. multilocularis* respectively
